# Linear Methods for Analysis and Quality Control of Relative Expression Ratios from Quantitative Real-Time Polymerase Chain Reaction Experiments

**DOI:** 10.1100/tsw.2011.124

**Published:** 2011-07-07

**Authors:** Robert B. Page, Arnold J. Stromberg

**Affiliations:** ^1^ Department of Biology Auburn University Montgomery Alabama USA; ^2^ Department of Statistics University of Kentucky Lexington USA

**Keywords:** amplification efficiency, endogenous reference gene, gene expression, linear regression, real-time quantitative reverse transcription PCR, relative expression ratio

## Abstract

Relative expression quantitative real-time polymerase chain reaction (RT-qPCR) experiments are a common means of estimating transcript abundances across biological groups and experimental treatments. One of the most frequently used expression measures that results from such experiments is the relative expression ratio (R_E_), which describes expression in experimental samples (i.e., RNA isolated from organisms, tissues, and/or cells that were exposed to one or more experimental or nonbaseline condition) in terms of fold change relative to calibrator samples (i.e., RNA isolated from organisms, tissues, and/or cells that were exposed to a control or baseline condition). Over the past decade, several models of R_E_ have been proposed, and it is now clear that endogenous reference gene stability and amplification efficiency must be assessed in order to ensure that estimates of R_E_ are valid. In this review, we summarize key issues associated with estimating R_E_ from cycle threshold data. In addition, we describe several methods based on linear modeling that enable researchers to estimate model parameters and conduct quality control procedures that assess whether model assumptions have been violated.

## INTRODUCTION

### Overview

Reverse transcription quantitative real-time polymerase chain reaction (RT-qPCR) is a method of quantifying transcript abundances that is routinely used to investigate expression in a small to moderate number of genes. In particular, RT-qPCR is frequently used to confirm microarray results and to investigate the expression of rare transcripts[1,2,3,4,5,6]. The primary strength of RT-qPCR is that its large dynamic range makes it well suited for quantifying low abundance transcripts and transcripts that vary widely in abundance between groups of interest[7,8,9]. Nevertheless, there are a number of difficulties associated with generating high-quality RT-qPCR data. Previous reviews have described many of these issues, including ensuring that RNA is of a sufficient quality and purity[10,11], the pros and cons of various approaches to generating cDNA via reverse transcription[7,10,12,13], the importance of proper sample storage[11], the need for careful primer design and assay validation[11], and the advantages and drawbacks to different detection chemistries[6,10,14,15]. However, comparatively little effort has been spent reviewing the issues associated with analysis and quality control of RT-qPCR data.

Over the past decade, a number of statistical and computational approaches to analyzing RT-qPCR data have been suggested in the literature, and it is now clear that applying objective statistical methods to RT-qPCR data poses several challenges[8,9,16,17,18]. In this review, we briefly describe the numerical data generated via most relative expression RT-qPCR experiments and then discuss several practical issues that researchers conducting relative expression RT-qPCR experiments are likely to face. In particular, we focus on using linear models for parameter estimation and the evaluation of assumptions that are inherent to the calculation relative expression ratios ($R_E$). We then conclude by summarizing the steps involved in processing relative expression RT-qPCR data, briefly discussing the issue of error propagation – how uncertainty in the parameters used to calculate $R_E$ affects the uncertainty of $R_E$ itself – and listing a set of general guidelines for the quality control and analysis of $R_E$ values.

## The Quantification Cycle: The Central Value of RT-qPCR

The basic strategy underlying RT-qPCR is to record the accumulation of fluorescent dyes that label a specific nucleic acid product or double-stranded DNA molecule throughout the course of a PCR. The amount of product yielded by a PCR approximates a logistic (i.e., sigmoidal) curve when it is plotted as a function of the number of reaction cycles completed (Fig. 1). Thus, setting a threshold within the exponential phase of the amplification curve and recording the number of fractional cycles required to eclipse this threshold provides a correlate to the initial amount of template known as the quantification cycle ($C_q$; lower $C_q$ values correspond to more starting template). However, while $C_q$ is the value of interest in the majority of RT-qPCR experiments, its determination requires exclusion of ground phase cycles (Fig. 1; also known as the background or baseline) and determination of where along the y-axis, within the exponential phase, the threshold should be placed. Determination of the baseline and threshold is usually handled by proprietary software that comes with real-time PCR hardware, and different systems use different methods for baseline and threshold determination. Because this review focuses on how to analyze $C_q$ values rather than how to ensure they are valid, we do not discuss baseline and threshold determination further and refer interested readers to Bustin and Nolan[10] and Adams[19] for discussions of when to adjust the baseline and threshold manually.

## The Relative Expression Quantification Strategy

There are two general approaches to conducting RT-qPCR experiments. The first, known as absolute quantification, is based on calibration to a standard curve generated from a known external source (e.g., recombinant DNA) that enables one to express data in terms of transcripts per biological unit (e.g., copies/µg of tissue). The second, known as relative quantification, describes expression in arbitrary units that are based on comparisons to a calibrator sample or a series of calibrator samples (e.g., RNA isolated from control or unmanipulated sources). Because the relative quantification approach makes fewer assumptions, is less labor intensive, and is sufficient for most applications, it is the method most frequently used in basic research and is thus the focus of this review.

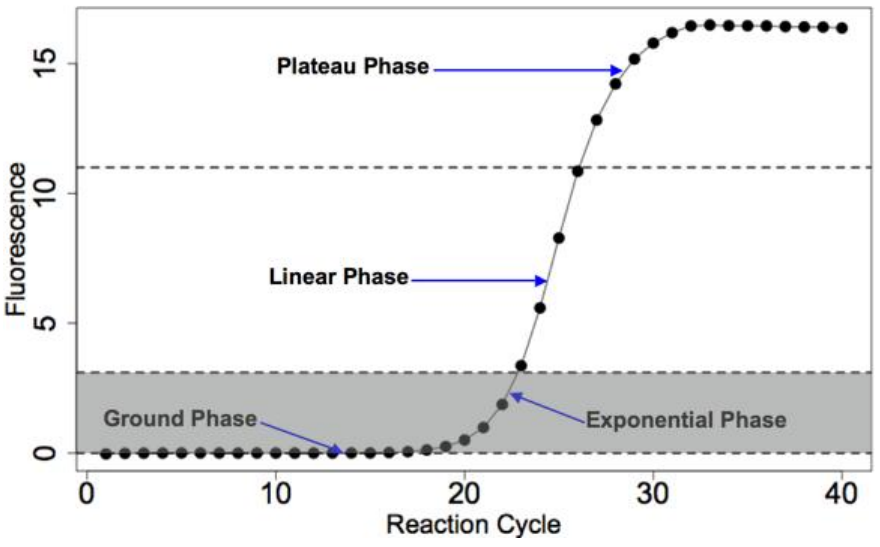

**FIGURE 1.** An example of a RT-qPCR amplification plot showing the four phases of a PCR. Dashed lines denote the approximate boundaries of each phase. The exponential phase is highlighted in gray. Note that the bottom dashed horizontal line corresponds to the ground phase.

The traditional approach to relative expression RT-qPCR is to plug the relevant $C_q$ values, or their averages, into one of a number of mathematical models that generate a $R_E$ describing expression in noncalibrator samples in terms of fold change relative to calibrator samples[8]. Usually, $R_E$ is normalized to one or more endogenous reference genes (ERGs)[8] because, in principle, this approach enables one to correct for variations in the amount and/or quality of the starting template that are introduced during upstream phases of the workflow[20]. The simplest and most widely used model of $R_E$ is known as the $2^{-\Delta\Delta Ct}$ method[21] and can be described by the following equations:

$$R_E = 2^{-[(GOIS-ERGS)-(GOIC-ERGC)]} \quad (1)$$

$$\Delta\Delta C_q = (GOIS - ERGS) - (GOIC - ERGC) \quad (2)$$

$$\Delta\Delta C_q = -\log_2(R_E) \quad (3)$$

where GOIS = $C_q$ for the gene of interest (GOI) from a noncalibrator sample, ERGS = $C_q$ for the ERG from a noncalibrator sample, GOIC = $C_q$ for the GOI from a calibrator sample, and ERGC = $C_q$ for the ERG from a calibrator sample. Although this model is popular due to its simplicity, it is based on a number of assumptions, some of which are more crucial to the inferential conclusions of a study than others (discussed in the next section). One of the most crucial assumptions is that the ERG being used for normalization is invariant across the groups being considered. Also of critical importance is that the reaction efficiencies are equal among the four reactions that are used to calculate $R_E$. Finally, there must be a doubling of the reaction product following every cycle (i.e., a percentile reaction efficiency [PAE] of one or a reaction efficiency [E] of two) in order for the $2^{-\Delta\Delta Ct}$ method to estimate the magnitude of $R_E$ accurately. In practice, any combination of these assumptions can be violated, with the end result being an inaccurate estimate of $R_E$ and/or spurious statistical significance[8,18,22,23].

## ADDRESSING ASSUMPTIONS THAT ARE LIKELY TO BE VIOLATED

### Endogenous Reference Gene Stability

Perhaps the most critical assumption of all relative expression RT-qPCR analyses is that the ERG used for normalization is invariant across all of the groups being considered[8,20,22,23]. It has long been known that assuming that highly expressed "housekeeping" genes are invariant across treatments/groups is poor practice. Moreover, it is unlikely that any genes are universally suitable as ERGs across all tissues and research paradigms. Therefore, it is important to verify that ERGs are invariant each time one wishes to investigate a new experimental system or tissue[7,14,23,24]. However, ERG validation is often challenging and can become a circular problem because without a way of accounting for the effect of each RNA sample, it can be difficult to tell whether differences in ERG $C_q$ values are due to differences among the groups being compared or to the technical variation that one is trying to remove via normalization[25,26,27].

A widely used approach to ERG validation is implemented by the geNorm software package[25]. Here, it is assumed that candidate ERGs are not coregulated, so that pair-wise calculations between the candidates based on the set of RNA samples to be compared can be used to arrive at metrics of stability for each candidate[25]. These metrics are, in turn, used to arrive at a subset of candidate ERGs from which a normalization factor (NF) based on the geometric mean of the subset is calculated. However, this so-called pair-wise approach has been criticized by Andersen et al.[26], who put forth a model-based approach to ERG validation that is implemented by the NormFinder software package. The statistical model proposed by Andersen et al.[26] is:

$$y_{igj} = \alpha_{ig} + \beta_{gj} + \varepsilon_{igj} \tag{4}$$

where $y_{igj}$ = the log-transformed expression measure for candidate ERG $i$ in the $j$th sample of group $g$, $\alpha_{ig}$ = the effect of candidate ERG $i$ within experimental group $g$, $\beta_{gj}$ = the effect of RNA sample $j$ from experimental group $g$, and $\varepsilon_{igj}$ = a random variable (error term) with a mean of zero and variance $\sigma^2$. By using this model as a starting point, Andersen et al.[26] arrived at estimates of intra- and intergroup variation in gene expression for candidate ERGs, and derived stability values (i.e., metrics) for each candidate. Andersen et al.[26] compared their approach to the pair-wise approach of Vandesompele et al.[25], and were able to show that their model-based approach selected ERGs with low intra- and intergroup variation and was robust to candidate panels in which some of the candidate ERGs were coregulated. The pair-wise approach, on the other hand, selected sets of ERGs with correlated expression profiles rather than genes with low intra- and intergroup variation. Thus, in situations where the expression of candidate ERGs are correlated across groups/treatments, the pair-wise method of Vandesompele et al.[25] may select ERGs that lead to inaccurate estimates of $R_E$[26].

Although the model-based approach of Andersen et al.[26] is robust with respect to candidate ERG panels in which some of the genes are coregulated, its validity depends on several assumptions. For example, the model-based approach assumes that the average expression of the candidate ERGs does not vary across the groups being considered. To meet this assumption, Andersen et al.[26] originally demonstrated the model-based approach with candidate ERGs that were carefully selected from microarray data that suggested that the candidates were stably expressed across the groups of interest. Thus, in cases where microarray data are not available for candidate ERG identification, it may be difficult to assess the assumptions of the model-based approach. Hence, when microarray data are not available ahead of time, the pair-wise method of Vandesompele et al.[25] may be more feasible, provided that the user is aware of its assumptions and limitations (see above).

To conclude our discussion on ERG stability, we point out that mixed effect models provide a well-established framework for modeling correlated data and individual effects[28]. Nevertheless, to the best of our knowledge, mixed models have not been used to derive statistical tests of ERG stability. However, if we wish to evaluate the suitability of a candidate ERG among *g* groups and *j* RNA samples, then provided that there is sufficient technical replication of $C_q$ for each of the *j* samples, we can formulate the following mixed model:

$$y_{igj} = \beta_0 + G_g + I_j(G_g) + \varepsilon_{igj} \qquad (5)$$

where $y_{igj}$ = the *i*th $C_q$ reading from the *j*th RNA sample in the *g*th group, $\beta_0$ = the intercept, $G_g$ = the fixed effect of the *g*th group, $I_j(G_g)$ = the random effect of the *j*th RNA sample nested within the *g*th group, and $\varepsilon_{igj}$ = the error associated with the *i*th $C_q$ reading from the *j*th RNA sample in the *g*th group. Of particular relevance is that this model allows us to avoid circularity by enabling us to assess whether there is significant variation in candidate ERG $C_q$ values among groups (i.e., test for statistical significance of the $G_g$ term) in the presence of parameters that allow for the statistical removal of the effects of individual RNA samples.

## Estimating Reaction Efficiency

Another assumption of the $2^{-\Delta\Delta Ct}$ model that is likely to be violated is the assumption of 100% reaction efficiency (i.e., E = 2 or PAE = 1) for all of the reactions that are used to calculate $R_E$[18]. While it is unlikely that any PCR has precisely 100% efficiency, implicit to this assumption is the additional assumption that all of the reactions used to calculate $R_E$ have equal efficiencies. Thus, while situations in which E differs from two, but is more-or-less equal among the reactions used to calculate $R_E$, will result in inaccurate estimates of $R_E$, they are not likely to result in erroneous inferences about differences between groups. However, cases in which the efficiencies of the reactions used to calculate $R_E$ are qualitatively different will lead to poor estimates of $R_E$ and may lead to erroneous inferences about differences between groups. Thus, the assumption of equivalent efficiencies is more critical than the assumption of 100% reaction efficiency when it comes to determining whether there are differences among groups. One of the first models to incorporate the concept of reaction efficiency into the calculation of $R_E$ was put forward by Pfaffl[29]. According to Pfaffl[29], the relative expression ratio is:

$$R_E = \frac{E_{GOI}^{(GOIC-GOIS)}}{E_{ERG}^{(ERGC-ERGS)}} \qquad (6)$$

where, $E_{GOI}$ = the reaction efficiency of the gene of interest, $E_{ERG}$ = the reaction efficiency of the endogenous reference gene, and GOIC, GOIS, ERGC, and ERGS are as defined above for Eq. 1. Recently, this model has been expanded by Hellemans et al.[30] to the following form that allows for multiple ERGs:

$$R_E = \frac{E_{GOI}^{(GOIC-GOIS)}}{\sqrt[f]{\prod_1^f E_{ERG}^{(ERGC-ERGS)}}} \qquad (7)$$

where *f* = the number of ERGs used for normalization and the remaining variables are as defined for Eq. 6. It is important to note that while these models account for differences in E between the GOI and ERG, they assume that neither $E_{GOI}$ nor $E_{ERG}$ vary between calibrator and noncalibrator samples. As is briefly discussed below, this assumption may be violated, and methods for assessing whether this is the case have been presented by Burns et al.[31] and Yuan et al.[17,18].

The introduction of E into relative quantification models means that E must be empirically estimated in order for these models to be used. The most commonly used approach for doing this is to estimate the average E from a series of reactions that were set up using a variety of cDNA template concentrations

(i.e., a dilution series). One can then equate the slope of the linear regression of $\log_{10}$(cDNA concentration) against $C_q$ to E as follows:

$$E = 10^{\left(-\frac{1}{m}\right)} \qquad (8)$$

where $m$ is the slope estimated via linear regression (Fig. 2A)[8,29]. More recently, Yuan et al.[17,18] suggested estimating the PAE by regressing the $\log_2$(cDNA concentration) against $C_q$ as in Fig 2B. E and PAE can be related by Eq. 9[18].

$$E = 2^{PAE} \qquad (9)$$

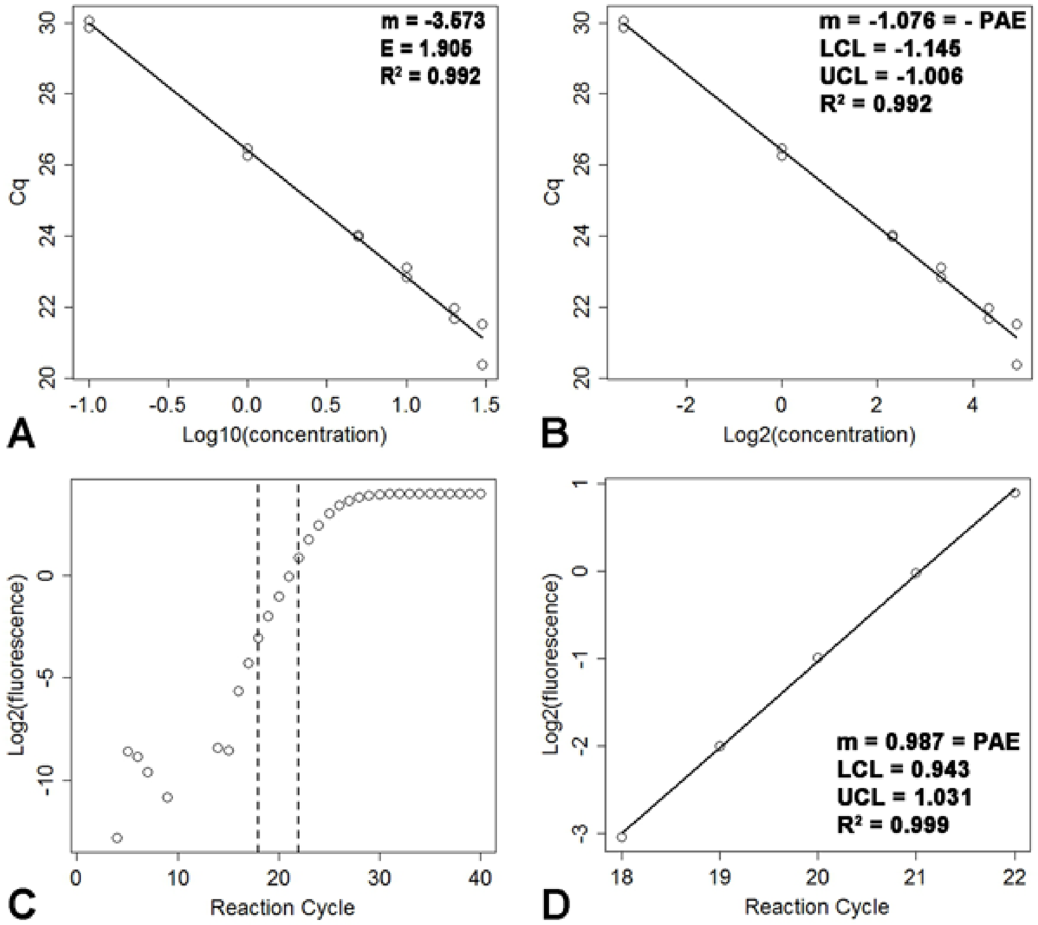

**FIGURE 2.** Various methods of estimating E and PAE. (A) Estimation of E from a dilution series using the $\log_{10}$(cDNA concentration) method. E is estimated using Eq. 8 (see text). (B) Estimation of PAE from the same dilution series shown in panel A using the $\log_2$(cDNA concentration) approach. LCL = lower 95% confidence limit of the slope and UCL = upper 95% confidence limit of the slope. Note that the estimate of PAE is marginally greater than 1 at the 0.05 level, indicating that this method has overestimated the true value of PAE. (C) A $\log_2$ transformation of the amplification plot shown in Fig. 1. The exponential phase of the reaction consists of cycles 18 through 22 as indicated by the dashed vertical lines. (D) Estimation of PAE using the exponential phase cycles highlighted in panel C. Note that the estimate of PAE is not significantly different from 1 at the 0.05 level.

The advantage of using the approach of Yuan et al.[17,18] is that the slope of the regression can be interpreted on the raw scale (i.e., PAE = -*m*, where *m* is the slope estimated via regression), which, in turn, leads to straightforward statistical tests of departures from 100% efficiency (i.e., is PAE = -1?) and differences among samples/groups (i.e., do the slopes estimated from different samples/groups differ from each other?; also see Burns et al.[31]).

Despite being the most commonly used method, estimating E or PAE from dilution series data has several drawbacks. Most obviously, the dilution series method requires considerable amounts of RNA and is laborious. Hence, for large experiments, it may not be feasible to estimate E for every sample and gene combination. In addition, the dilution series approach does not estimate reaction-specific efficiencies, but rather the average E across several reactions. Thus, dilution series–based estimations provide no means of identifying reactions with outlying E values (see below). Finally, dilution series–based methods occasionally yield estimates of E that are >2, suggesting that they are prone to overestimating E (Fig. 2B)[8].

The second general method for estimating reaction efficiencies is to use the cycle-by-cycle fluorescence data that are collected during the course of a real-time PCR (Fig. 1). This approach has the advantage of being able to yield an estimate of the reaction efficiency for every reaction. Furthermore, unlike the dilution series approach, it does not require additional labor as fluorescence data are acquired during the course of conducting an experiment. A number of strategies for estimating reaction efficiencies from fluorescence data have been suggested in the literature. However, the most straightforward approaches involve identifying the exponential phase of the amplification curve (Fig. 1; see Pierson et al.[32] and Ramakers et al.[33] for descriptions of algorithms that are useful for automating the process of identifying the exponential phase), and regressing the resulting $\log_{10}$[32,33] or $\log_2$[18] transformed subset of fluorescence values against cycle number (Fig. 2C,D). The resulting slope of this regression can be used to obtain an estimate of E or PAE (Fig 2D). As is the case with dilution series data, using the $\log_2$ scale has the advantage of yielding estimates of PAE that are interpretable on the raw scale as the slope of the regression on the $\log_2$ scale is itself an estimate of PAE.

While estimating reaction efficiencies from fluorescence data has several advantages over the dilution series method (see above), there are some drawbacks. An obvious concern is that for large experiments involving thousands of reactions, using florescence-based approaches creates a considerable informatics problem. Another concern that arises when using fluorescence-based methods that rely on linear regression is that the reaction efficiency estimates will be based on small sample sizes due to the exclusion of a large number of reaction cycles. Finally, it is not clear that using the efficiency estimates generated for every reaction in a dataset is the most appropriate use of this information as analyzing data based on reaction-specific efficiencies may introduce considerable noise into a dataset[32,34]. Pierson et al.[32] and Cikos et al.[34] have suggested that analyses based on averaged efficiencies provide more robust results and that reaction-specific efficiencies should be used primarily to exclude reactions that have outlying efficiencies.

In conclusion to our discussion of efficiency, we note that we have only reviewed strategies that use linear regression to estimate E or PAE from fluorescence data. Our reason for doing this is that linear regression is the most conceptually simple approach to estimating reaction efficiencies and is therefore likely to be the most accessible to practicing biologists with a limited background in statistics. Nevertheless, several strategies for addressing the issue of reaction efficiency that rely on nonlinear regression have also been proposed[35,36,37], many of which are implemented by the qpcR software package[38].

## STATISTICAL INFERENCE

### Conventional Tests and Data Transformation

By far the most common way in which RT-qPCR data are analyzed is via the use of standard parametric statistical tests (i.e., t-test, ANOVA, etc.) that assess whether $R_E$ varies as a function of the groups/treatments being considered. As described in the previous section, there are a number of situations that can render such analyses invalid. Nevertheless, if the assumptions that are essential to calculating unbiased estimates of $R_E$ are met, the application of objective statistical methods to relative expression RT-qPCR data is valid. When using conventional parametric statistics to evaluate RT-qPCR data, it is important to bear in mind that $R_E$ is not symmetrically scaled as up-regulated values of $R_E$ lie on one scale ($1 < R_E < \infty$) and down-regulated values lie on another scale ($1 > R_E > 0$). We recommend analyzing $\log_2(R_E)$ because log transformations of $R_E$ result in symmetrically scaled expression measures that are more likely to meet the assumption of normality that is inherent to most parametric models[27]. As shown in Eq. 3, $\Delta\Delta C_q$ lies on the same scale as $\log_2(R_E)$ and thus offers the same advantages.

## Permutation Procedures and Linear Models for Comparisons of Two Groups

Although most analyses of relative expression RT-qPCR data use conventional parametric tests, several authors have suggested RT-qPCR–specific methods for drawing inferences about whether $R_E$ (or transformations thereof) statistically differs between two groups of interest. One of the earliest methods for doing this was put forward by Pfaffl et al.[39] and is based on a resampling procedure. In this approach, two biologically replicated groups (i.e., calibrator and noncalibrator) are considered in which one group consists of $n_1$ samples and the second group consists of $n_2$ samples. For each sample in both groups, GOI and ERG $C_q$ values are generated (potentially with technical replication) and $R_E$ is calculated according to Eq. 6, using the means of the two respective groups (i.e., calibrator and noncalibrator) for GOIS, ERGS, GOIC, and ERGC. A large number of pseudosamples (>1000) are then generated by permutating the group labels of the GOI and ERG readings. $R_E$ is then calculated for each pseudosample and the proportion of pseudosamples with more extreme $R_E$ values than the observed $R_E$ value is used to estimate a $p$ value for the null hypothesis that the two groups have an $R_E$ of 1 (i.e., $\log[R_E] = 0$; see Pfaffl et al.[39] for additional details).

More recently, Yuan et al.[17,18] proposed a number of ways to estimate and assess the statistical significance of $\Delta\Delta C_q$ using linear models. Particularly noteworthy is that Yuan et al.[18] were able to demonstrate the flexibility of their approach by presenting methods for estimating an efficiency-adjusted $\Delta\Delta C_q$ ($\Delta\Delta Cq_{adj}$; Eq. 10) based on dilution series data and fluorescence data.

$$\Delta\Delta Cq_{adj} = [(GOIS)(PAE_{GOIS}) - (ERGS)(PAE_{ERGS})] - [(GOIC)(PAE_{GOIC}) - (ERGC)(PAE_{ERGC})] \qquad (10)$$

While a comprehensive review of the methodologies developed by Yuan et al.[17,18] for estimating and assessing $\Delta\Delta C_q$ is beyond the scope of this review, we present one of the models from Yuan et al.[17] to give a feel for these authors' approach to assessing statistical significance of RT-qPCR data. Here, we assume that a researcher wants to assess whether two biologically replicated groups (i.e., calibrator and noncalibrator) differ in the expression of a GOI that has been normalized to an ERG. Moreover, we assume that the researcher has generated dilution series data for every calibrator and noncalibrator sample for both the GOI and the ERG. Once in place, these data can be recoded into four groups such that group one corresponds to GOI readings from calibrator samples, group two corresponds to GOI readings from noncalibrator samples, group three corresponds to ERG readings from calibrator samples, and group four corresponds to ERG readings from noncalibrator samples. An analysis of covariance (ANCOVA) model of the following form can then be fit to the data:

$$C_q = \beta_0 + \beta_{con}X_{icon} + \beta_{group}X_{igroup} + \beta_{groupcon}X_{igroup}X_{icon} + \varepsilon \qquad (11)$$

where $\beta_0$ = the intercept, $\beta_{con}$ = the effect of template concentration, $\beta_{group}$ = the effect of the grouping variable described above, $\beta_{groupcon}$ = the effect of the interaction between group and concentration, and $\varepsilon$ = the error term. It then follows that contrasting the parameters associated with the $\beta_{group}$ term (i.e., $\mu_x$ below where x is one of the four groups described above) according to Eq. 12 results in an estimate of $\Delta\Delta C_q$ as well as a test of the null hypothesis that $\Delta\Delta C_q = 0$.

$$(\mu_2 - \mu_1) - (\mu_4 - \mu_3) = 0 \quad (12)$$

The parameters associated with the $\beta_{groupcon}$ term are also of interest as they are slopes that describe how $C_q$ changes as a function of $\log_2$(template concentration) for each of the four groups. Thus, the $\beta_{groupcon}$ parameters can be used to test for equal efficiencies among groups and/or to efficiency correct (see Eq. 10) the $\Delta\Delta C_q$ estimate that results from the contrast presented in Eq. 12[18].

While the RT-qPCR specific approaches to inference described above provide explicit statistical frameworks for interpreting results, they do not, in and of themselves, alleviate the burden of meeting the assumptions of relative expression models. Therefore, it is still critical to ensure that ERGs are invariant and that data comply with the assumptions about reaction efficiency made by the model. At present, a major drawback to many of the RT-qPCR specific statistical tests is that they only allow for comparisons of two groups. Thus, there is a need for general RT-qPCR–specific statistical frameworks that enable researchers to assess complex experimental designs, while simultaneously providing tools for inspecting data quality with respect to the assumptions that are critical to the validity of the method.

## DATA PROCESSING, ERROR PROPAGATION, AND RECOMMENDATIONS

It has now been repeatedly demonstrated that the way in which RT-qPCR data are processed and analyzed can strongly influence the biological conclusions drawn from the data[16,40]. Although a large number of processing procedures have been described in the literature, there is currently no consensus on which methods are most robust. The quality control and processing steps that are essential to calculating valid $R_E$ values include baseline determination, threshold determination, control gene validation, efficiency estimation, and removal of reactions with outlying $C_q$[31] and/or E values. In addition, if samples from separate runs (i.e., separate microtiter plates) are to be directly compared, it is likely that some sort of inter-run calibration will be required[30]. One of the most important issues associated with data processing that is a source of ongoing research is the issue of error propagation[9,30,41]. Of particular concern within the context of the approaches to data analysis discussed in this review is that all of the components needed to calculate $R_E$, such as efficiency estimates and $C_q$ values, are themselves measured with uncertainty. Although it is common practice to technically replicate $C_q$ values (i.e., taking the average of several $C_q$ values that were generated from the same RNA sample), the dispersion estimates (e.g., variance, standard error, etc.) associated with technically replicated $C_q$ values are often not used to calculate measures of dispersion for $R_E$. Moreover, error propagation with respect to the uncertainty surrounding efficiency estimates is also frequently lacking. Thus, there is a need to develop processing procedures that account for this uncertainty as well as user-friendly software implementations of these procedures that make them readily available to practicing biologists.

When conducting RT-qPCR experiments, one of the first decisions that must be made is which genes to evaluate for suitability as ERGs. In cases where companion microarray data are available, the model-based approach of Andersen et al.[26] is well suited for ERG identification. In situations where companion microarray data are not available, the pair-wise approach of Vandesompele et al.[25] may enable the identification of ERGs that allow for the calculation of a stable NF. Irrespective of whether a single ERG or an NF is used for normalization, the mixed model presented in Eq. 5 can be used as a *post hoc* assessment of whether the selected ERG or NF is stable across the groups of interest after the effects of the individual RNA samples have been statistically removed. Once it is clear that the ERG/NF is indeed stable, the next step is to estimate E or PAE. In our opinion, estimating the PAE from fluorescence data on the $\log_2$ scale according to Yuan et al.[18] offers several advantages. First, unlike the dilution series method, it does not require additional labor, reagents, and RNA, which may be prohibitive if there are a large number of samples or GOIs to investigate. Second, linear regression on the $\log_2$ scale enables a direct estimate of PAE for every reaction as well as a statistical test of whether PAE statistically differs from one. Third, standard methodologies for comparing the slopes of different regression models[31,42] can be used to assess whether any of the slopes used to calculate a given $R_E$ value statistically differ from one another. Therefore, one is not forced to make the assumption that $E_{ERG}$ and $E_{GOI}$ are equal across the groups being considered (see Eqs. 6 and 7). Eq. 10 can then be used to calculate $\Delta\Delta Cq_{adj}$. However, the PAE parameters in this equation should be based on the average PAE for each of the four respective groups, and reactions with outlying $C_q$ values and/or PAE values should be excluded. In cases where there are more than two groups to compare, there will be more than one noncalibrator group. Because $\Delta\Delta Cq_{adj}$ lies on the $\log_2$ scale, $\Delta\Delta Cq_{adj}$ can itself be treated as an expression measure and analyzed using conventional parametric statistical tests such as ANOVA.

## ACKNOWLEDGMENTS

We would like to thank Chi Wang, Anthony Athippozhy, and two anonymous referees for critical reviews that improved the content of this manuscript. This work was supported by funding from NIH P20 RR16481 to AJS.

## References

1. Rajeevan, M.S., Ranamukhaarachchi, D.G., Vernon, S.D., and Unger, E.R. (2001) Use of real-time quantitative PCR to validate the results of cDNA array and differential display PCR technologies. *Methods* **25**, 443–451.
2. Chuaqui, R.F., Bonner, R.F., Best, C.J.M., Gillespie, J.W., Flaig, M.J., Hewitt, S.M., Phillips, J.L., Krizman, D.B., Tangrea, M.A., Ahram, M., Linehan, W.M., Knezevic, V., and Emmert-Buck, M.R. (2002) Post-analysis follow-up and validation of microarray experiments. *Nat. Genet. Suppl*. **32**, 509–514.
3. Rockett, J.C. and Hellmann, G.M. (2004) Confirming microarray data—is it really necessary? *Genomics* **83**, 541–549.
4. Canales, R.D., Luo, Y., Willey, J.C., Austermiller, B., Barbacioru, C.C., Boysen, C., Hunkapiller, K., Jensen, R.V., Knight, C.R., Lee, K.Y., Ma, Y., Maqsodi, B., Papallo, A., Peters, E.H., Poulter, K., Ruppel, P.L., Samaha, R.R., Shi, L., Yang, W., Zhang, L., and Goodsaid, F.M. (2006) Evaluation of microarray results with quantitative gene expression platforms. *Nat. Biotechnol.* **24**, 1115–1122.
5. Draghichi, S., Khatri, P., Eklund, A.C., and Szallasi, Z. (2006) Reliability and reproducibility issues in DNA microarray measurements. *Trends Genet*. **22**, 101–109.
6. VanGuilder, H.D., Vrana, K.E., and Freeman, W.M. (2008) Twenty-five years of quantitative PCR for gene expression analysis. *Biotechniques* **44**, 619–626.
7. Bustin, S.A., Benes, V., Nolan, T., and Pfaffl, M.W. (2005) Quantitative real-time RT-PCR – a perspective. *J. Mol. Endocrinol*. **34**, 597–601.
8. Pfaffl, M.W. (2006) Relative quantification. In *Real-time PCR*. Dorak, M.T., Ed. Taylor and Francis, New York. pp. 63–82.
9. Karlen, Y., McNair, A., Perseguers, S., Mazza, C., and Mermod, N. (2007) Statistical significance of quantitative PCR. *BMC Bioinformatics* **8**, 131.
10. Bustin, S.A. and Nolan, T. (2004) Pitfalls of quantitative real-time reverse transcription polymerase chain reaction. *J. Biomol. Tech*. **15**, 155–166.
11. Bustin, S.A., Benes, V., Garson, J.A., Hellemans, J., Huggett, J., Kubista, M., Mueller, R., Nolan, T., Pfaffl, M.W., Shipley, G.L., Vandesompele, J., and Wittwer, C.T. (2009) The MIQE guidelines: minimum information for publication of quantitative real-time PCR experiments. *Clin. Chem*. **55**, 611–622.
12. Bustin, S.A. (2000) Absolute quantification of mRNA using real-time reverse transcription polymerase chain reaction assays. *J. Mol. Endocrinol*. **25**, 169–193.
13. Bustin, S.A. (2002) Quantification of mRNA using real-time reverse transcription PCR (RT-PCR): trends and problems. *J. Mol. Endocrinol*. **29**, 23–39.
14. Wong, M.L. and Medrano, J.F. (2005) Real-time PCR for mRNA quantification. *Biotechniques* **39**, 75–85.
15. Shipley, G.L. (2006) An introduction to real-time PCR. In *Real-time PCR*. Dorak, M.T., Ed. Taylor and Francis, New York. pp. 1–31.
16. Skern, R., Frost, P., and Nilsen, F. (2005) Relative transcript quantification by quantitative PCR: roughly right or precisely wrong. *BMC Mol. Biol.* **6**, 10.
17. Yuan, J.S., Reed, A., Chen, F., and Stewart, C.N. (2006) Statistical analysis of real-time PCR data. *BMC Bioinformatics* **7**, 85.
18. Yuan, J.S., Wang, D., and Stewart, C.N. (2008) Statistical methods for efficiency adjusted real-time PCR

quantification. *Biotechnol. J.* **3**, 112–123.
19. Adams, P.S. (2006) Data analysis and reporting. In *Real-time PCR*. Dorak, M.T., Ed. Taylor and Francis, New York. pp. 39–62.
20. Huggett, J., Dheda, K., and Bustin, S.A. (2006) Normalization. In *Real-time PCR*. Dorak, M.T., Ed. Taylor and Francis, New York. pp. 83–91.
21. Livak, K.J. and Schmittgen, T.D. (2001) Analysis of relative gene expression data using real-time quantitative PCR and the $2^{-\Delta\Delta Ct}$ method. *Methods* **25**, 402–408.
22. Dheda, K., Huggett, J.F., Bustin, S.A., Johnson, M.A., Rook, G., and Zumla, A. (2004) Validation of housekeeping genes for normalizing RNA expression in real-time PCR. *Biotechniques* **37**, 112–119.
23. Gutierrez, L., Mauriat, M., Guenin, S., Pelloux, J., Lefebvre, J.F., Louvet, R., Rusterucci, C., Moritz, T., Guerineau, F., Bellini, C., and Van Wuytswinkel, O. (2008) The lack of a systematic validation of reference genes: a serious pitfall undervalued in reverse transcription-polymerase chain reaction (RT-PCR) analysis in plants. *Plant Biotechnol. J.* **6**, 609–618.
24. Huggett, J., Dheda, K., Bustin, S.A., and Zumla, A. (2005) Real-time RT-PCR normalisation; strategies and considerations. *Genes Immun.* **6**, 279–284.
25. Vandesompele, J., De Preter, K., Pattyn, F., Poppe, B., Van Roy, N., De Paepe, A., and Speleman, F. (2002) Accurate normalization of real-time quantitative RT-PCR data by geometric averaging of multiple internal control genes. *Genome Biol.* **3**, Research0034.
26. Andersen, C.L., Jensen, J.L., and Orntoft, T.F. (2004) Normalization of real-time quantitative reverse transcription-PCR data: a model-based variance estimation to identify genes suited for normalization, applied to bladder and colon cancer data sets. *Cancer Res.* **64**, 5245–5250.
27. Pfaffl, M.W., Tichopad, A., Prgomet, C., and Neuvians, T.P. (2004) Determination of stable housekeeping genes, differentially regulated target genes and sample integrity: BestKeeper – Excel-based tool using pair-wise correlations. *Biotechnol. Lett.* **26**, 509–515.
28. Jiang, J. (2007) *Linear and Generalized Linear Mixed Models and Their Applications*. Springer, New York.
29. Pfaffl, M.W. (2001) A new mathematical model for relative quantification in real-time RT-PCR. *Nucleic Acids Res.* **29**, e45.
30. Hellemans, J., Mortier, G., De Paepe, A., Speleman, F., and Vandesompele, J. (2007) qBase relative quantification framework and software for management and automated analysis of real-time quantitative PCR data. *Genome Biol.* **8**, R19.
31. Burns, M.J., Nixon, G.J., Foy, C.A., and Harris, N. (2005) Standardisation of data from real-time quantitative PCR methods-evaluation of outliers and comparison of calibration curves. *BMC Biotechnol.* **5**, 31.
32. Pierson, S.N., Butler, J.N., and Foster, R.G. (2003) Experimental validation of novel and conventional approaches to quantiative real-time PCR data analysis. *Nucleic Acids Res.* **31**, e73.
33. Ramakers, C., Ruijter, J.M., Deprez, R.H.L., and Moorman, A.F.M. (2003) Assumption-free analysis of quantitative real-time polymerase chain reaction (PCR) data. *Neurosci. Lett.* **339**, 62–66.
34. Cikos, S., Bukovska, A., and Koppel, J. (2007) Relative qunatification of mRNA: comparison of methods currently used for real-time PCR data analysis. *BMC Mol. Biol*. **8**, 113.
35. Liu, W. and Saint, D.A. (2002) Validation of a quantitative method for real time PCR kinetics. *Biochem. Biophys. Res. Commun.* **294**, 347–353.
36. Tichopad, A., Dilger, M., Schwarz, G., and Pfaffl, M.W. (2003) Standardized determination of real-time PCR efficiency from a single reaction set-up. *Nucleic Acids Res.* **31**, e122.
37. Rutledge, R.G. (2004) Sigmoidal curve-fitting redefines quantitative real-time PCR with the prospective of developing automated high-throughput applications. *Nucleic Acids Res.* **32**, e178.
38. Ritz, C. and Spiess, A.N. (2008) qpcR: an R package for sigmoidal model selection in quantitative real-time polymerase chain reaction analysis. *Bioinformatics* **24**, 1549–1551.
39. Pfaffl, M.W., Horgan, G.W., and Dempfle, L. (2002) Relative expression software tool (REST) for group-wise comparison and statistical analysis of relative expression results in real-time PCR. *Nucleic Acids Res.* **30**, e36.
40. Bohlenius, H., Eriksson, S., Parcy, F., and Nilsson, O. (2007) Retraction. *Science* **316**, 367.
41. Larionov, A., Krause, A., and Miller, W. (2005) A standard curve based method for relative real time PCR data processing. *BMC Bioinformatics* **6**, 62.
42. Sokal, R.R. and Rohlf, F.J. (1995) *Biometry*. 3rd ed. Freeman, New York.
